# When Pushing the Access “Easy Button” Gets Complicated: Troubleshooting Transcarotid TAVR Access Issues

**DOI:** 10.1016/j.jaccas.2026.109330

**Published:** 2026-08-26

**Authors:** Andrei Pop

**Affiliations:** Alexian Brothers Medical Center, Elk Grove Village, Illinois, USA

**Keywords:** aortic valve, atherosclerosis, valve replacement

**Figure:**
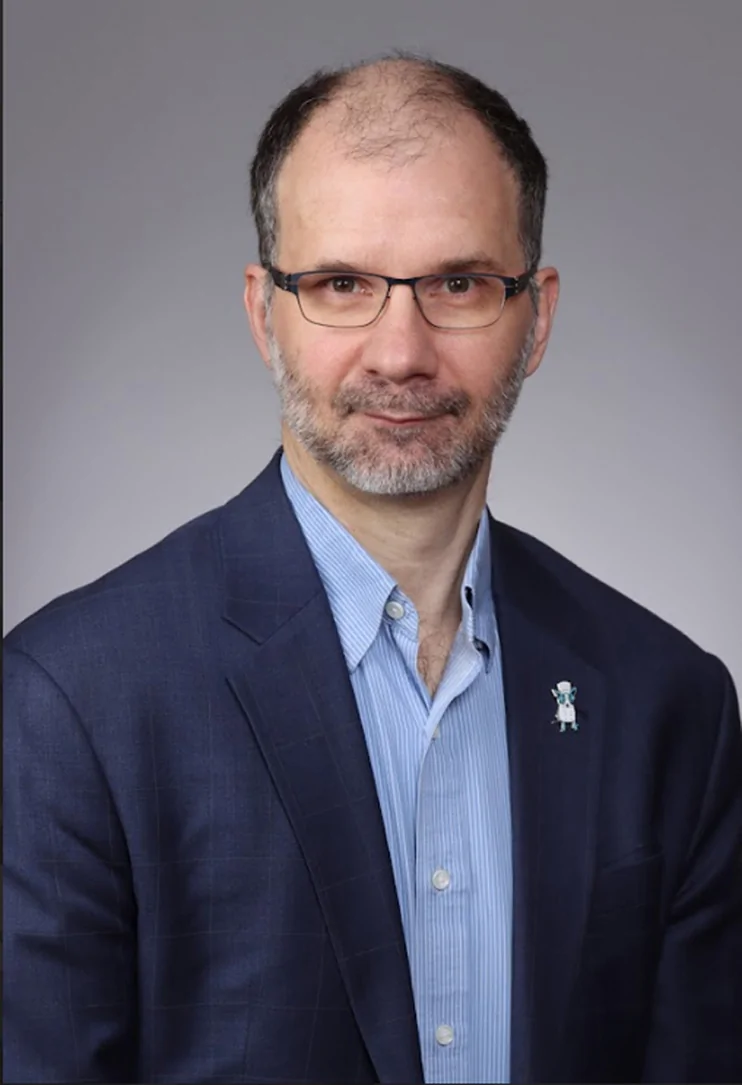

Transcarotid (TC) transcatheter aortic valve replacement (TAVR) access, first described in 2009,^1^ has rapidly become the preferred alternative TAVR access route in the United States.^2^ TC access brings several advantages, including proximity to the aortic valve, extra-thoracic access, predictable closure, and low stroke rates. A small minority of patients are deemed unsuitable for TC access, often due to extensive cerebrovascular disease or prior neck irradiation. TC access is associated with lower vascular complication rates vs transcaval^3^ and a lower stroke rate vs transaxillary/subclavian.^4^ The widespread adoption of TC access, together with the lower size of TAVR sheaths and a lower complication rate have rendered transaortic and transapical access extremely rare in contemporary practice.^5^

TC access with the Sapien system can be performed using several different sheath systems, each with their respective advantages/disadvantages. The standard Commander Delivery system and 14/16-F e-sheath is most familiar to operators but can be unwieldly in a TC approach due to its length. Unfortunately, because of the need for valve mounting inside the body, if the aortic valve cannot be easily crossed, the mounted Sapien valve cannot be withdrawn in the e-sheath. The Certitude system, designed for transapical access, permits the insertion of the already mounted valve, eliminating the need for mounting of the valve on the balloon in the ascending aorta. The large-diameter sheath (18- and 21-F) and lack of a hydrophilic coating can make use of this system challenging in smaller carotid arteries. Furthermore, although the system continues to be available in the United States, it is no longer available in other markets. Insertion of the mounted valve through a large Gore Dry-Seal stealth also can be considered. The large diameter of the sheath required (20-F for a 20 S3 Ultra to 26-F for a 29 S3 Ultra valve) renders this approach applicable only to a minority of cases.

The management of complications during TC access can be challenging. Balloon rupture, valve embolization, and coronary occlusion troubleshooting steps are less well characterized during TC than during transfemoral access. The fact that TC access is used in cases where transfemoral access would be challenging also means that a second large-bore access site is generally not available for bailout in case of complications.

In this issue of the journal, Miyamoto et al^6^ describe the use of a buddy-balloon technique to facilitate delivery of a 26-mm S3 Ultra Resilia valve in a calcified anatomy. The authors are to be applauded for their success in a challenging situation in which attempts at valve delivery resulted in hemodynamic instability in an older patient. The case highlights the need for comprehensive contingency planning in all TAVR cases—transfemoral as well as alternative access. Even though TC access has been selected for valve delivery, other access sites need to be examined during procedural planning to assess their suitability for use should bailout become necessary. The lack of another standard large-bore access site makes it essential to prospectively identify and have available bailout equipment that can be safely inserted through smaller-diameter sheaths. This case also highlights the importance of interventional techniques—even in TC access cases, as well as the importance of adequate predilation, especially when withdrawing the mounted valve is not feasible. Vascular complications that can be encountered during TC access are outlined in Table 1, together with prevention and management steps. Although the interventional cardiologist may be perceived as playing a lesser role in TC TAVR, this case is a perfect illustration of why the multidisciplinary team approach remains essential.

**Table 1:** Prevention and Management of Valvular Complications During Transcarotid TAVR With Sapien

Issue	Prevention	Management
Inability to cross	Adequate predilation	Predilation from separate access once TAVR has been introduced and mounted.
		Valve delivery balloon tip inflation.
		Buddy wire/balloon from separate access.
		Snaring of delivery system nose-cone in left ventricle.
Balloon rupture	Predilation	Longitudinal rupture: Remove from carotid access.
		Circumferential rupture: Snare from same or separate access and attempt percutaneous removal. Surgical cutdown and removal as bailout if percutaneous removal fails.
Valve embolization	Adequate sizing, effective pacing	Secure valve in ascending aorta if vessel diameter permits it.
		Place wire through the valve from femoral access and secure valve in descending aorta if ascending aorta too large to secure valve there.

TAVR = transcatheter aortic valve replacement.

## Funding Support and Author Disclosures

Dr Pop has been a consultant for Edwards Lifesciences and Inari Medical.
